# Management of Grade IV Pressure Ulcers With a Novel Negative Pressure Device in Traumatic Paraplegia Subjects

**DOI:** 10.7759/cureus.9327

**Published:** 2020-07-21

**Authors:** Mukesh K Dwivedi, Amit Bhagat, Rajeshwar N Srivastava, Lavini Raj

**Affiliations:** 1 Orthopaedic Surgery, King George's Medical University, Lucknow, IND; 2 Data Analytics, Hult International Business School, San Francisco, USA; 3 Medical Physics, King George's Medical University, Lucknow, IND

**Keywords:** pressure ulcer, negative pressure wound therapy, negative pressure device

## Abstract

Introduction

Pressure ulcers (PUs) are a major health problem for bedridden patients or persons with reduced mobility; individuals with spinal cord injury (SCI) are more prone to developing pressure ulcers. The purpose of this study was to determine the efficacy of a novel negative pressure wound therapy (NPWT) system for the treatment of Grade IV PUs.

Methods

A total of 34 SCI patients with Grade IV PUs were divided into two groups: 17 cases were managed by our bellows-powered negative pressure device (NPD) and 17 received wet-to-moist gauze dressing as standard wound care.

Results

Wound healing outcome measures were recorded every week (at seven days) and compared at weeks 3, 6, and 9. There were no significant changes in the length and width of PUs between the groups till week 3. Significantly reduced length and width of NPD-treated PUs were found at week 6 (p=0.04) that further reduced at week 9 (p=0.001) as compared to standard wound care. Similarly, significant reduction in the depth of PUs was found in the NPD-treated group at week 9 (p<0.05). Exudate levels were significantly (p=0.001) lower in the NPD-treated group as compared to the standard wound care group from week 3 (2.96±0.21 vs 2.62±0.49); this difference continued through week 9 (1.35±0.75 vs 0.14±0.35). Disappearance of slough and formation of healthy granulation tissue was significantly higher in the NPD-treated PUs after week 6 (p=0.001).

Conclusion

NPWT may be the future of wound healing. As an alternative to the existing electrically powered NPWT systems, our NPD is safe, easy to apply, and efficacious in treating the PUs.

## Introduction

Pressure ulcers (PUs) are wounds initiated by pressure on the skin that blocks circulation, causing the skin and underlying tissues to die [1,2]. PUs are a major health problem for bedridden patients with reduced mobility; patients with spinal cord injury (SCI) and associated co-morbidities are more prone to developing PUs with the incidence of 25%-66% [3-6]. Chronic wounds such as PUs are difficult to treat with available standard medical therapy and represent a burden on health care systems and also have a socio-economic impact on the patients and their caregivers. Developing more effective treatments is therefore a necessity [7,8].

Negative pressure wound therapy (NPWT) includes a mechanical, vacuum-assisted method that exerts negative pressure of −125 mm Hg on the wound bed [9,10]. The mechanism by which NPWT promotes wound healing is fairly clear. It is believed that the negative pressure promotes wound healing by the removal of interstitial fluid along with matrix metalloproteinases, decreasing edema, increasing blood flow, and formation of new blood vessels, thereby supplying the oxygen, growth factors, and nutrition to the wounds. Also, the mechanical deformation of cells is thought to result in protein and matrix molecule synthesis, which increases the rate of cell proliferation and granulation tissue formation, which in turn may promote healing [11-13].

NPWT is evolving and is under investigation for the management of difficult, chronic, and unrelenting wounds. The available negative pressure devices (NPDs) for NPWT are expensive and hard-to-afford by patients and health systems in developing countries. As an alternative to these NPWT systems, we propose a bellows-powered NPD for the management of PUs. The purpose of this study was to assess healing outcome measures such as surface area and depth, levels of exudate, and formation of granulation tissue in patients treated with our NPD compared to patients managed by wet-to-moist gauze dressings.

## Materials and methods

This study was conducted at the Spinal Cord Injury Unit, Department of Orthopaedic Surgery, King George's Medical University, Lucknow, India. The study was approved by the Institutional Ethics Committee (IEC) of the University (KGMU). In this prospective non-randomized study, 34 SCI patients with PUs of Grade IV, according to the National Pressure Ulcer Advisory Panel (NPUAP), were recruited [14]. Out of 34 patients, 17 were managed by our novel NPD and the rest were managed by wet-to-moist gauze dressing as standard wound care. We allocated participants to either group without attempting randomization. The additional inclusion criteria were SCI patients of age 16-45 years of either gender. Exclusion criteria were PUs with necrotic tissue that could not be removed on baseline debridement, chronic osteomyelitis, exposed blood vessels, and nerves in the wound. We also excluded patients undergoing chemotherapy or radiation therapy and persons with severe nutritional deficits (Braden Scale for Pressure Sore Risk - Nutrition subscale score of ≤2, a serum albumin level of <2.5 g/L, or a hemoglobin level of <9.0 g/L). The study protocol was explained to patients in their local language and informed consent was obtained.

Baseline assessment of ulcers

Information regarding patient demographics, PU history, and co-morbidities was obtained from patients and/or their carers. Wound debridement for necrotic tissue and slough was done in all patients before being allocated to either group. The allocation of patients was done by the primary author throughout the study period. The PUs were measured for their length and width with a centimeter ruler. The surface area was calculated from these values. PUs in both groups were measured at each time point (weekly) using the uniform procedure. PU depth was measured with a sterilized cotton-tipped applicator, which was inserted into the ulcer and marked at the deepest level. The amount of exudate was categorized as none (0), light (1), moderate (2), or heavy (3) after the dressing was removed in both NPWT and Standard Care groups with the help of the Pressure Ulcer Scale for Healing (PUSH) tool, version 3.0 (NPUAP, Westford, MA) [15]. Necrotic tissue, slough, and formation of red granulation tissue were assessed by visual inspection at the time of dressing change in both groups. Weekly assessment of PUs for every outcome measure and clinical photography was carried out by a co-author throughout the study. The actual cost of all consumables required for NPWT by our NPD and for standard care was calculated for two representative PUs of similar size in each group. The patients were followed up for nine weeks. Data were recorded every seven days and analysed after completion of a nine-week follow-up.

Method of standard wound care

The surface of the PU was cleansed with normal saline and packed with sterilized gauze to cover the wound. Dressing changes were performed once or twice daily depending on the soakage of the dressing.

Components of the novel NPD

The novel NPD was applied exclusively as a bedside procedure. It is a low-cost device and comprises a low-power continuous suction apparatus consisting of the following: a bellow unit of 800 mL capacity, a connecting tube with clamp, a "Y" connector, a curved needle with a matching catheter and a spare perforated catheter (Romo Vac Set® GS-5002 size-10; Romsons Scientific & Surgical Industries Pvt Ltd, Agra, UP, India); a sterilized piece of foam; a transparent polyurethane adhesive dressing (Opsite; G. Surgiwear Ltd, Shahjahanpur, UP, India); and a Dynaplast elastic adhesive bandage (Johnson & Johnson India Ltd, Mumbai), which sealed the adhesive dressing to the adjoining skin. We assembled these components to form a novel NPD that was applied to the PU and changed every week or earlier if there was a soakage/leakage. The cost of our NPD was obtained from the record of the hospital's central supply department. The cost of the required components of our NPD was about 40-50 USD. A single Romo Vac Set and a Dynaplast were utilized throughout the nine weeks, while one or more transparent polyurethane dressing and foam were used during the nine-week follow-up.

Application of the novel NPD

**Figure 1:**
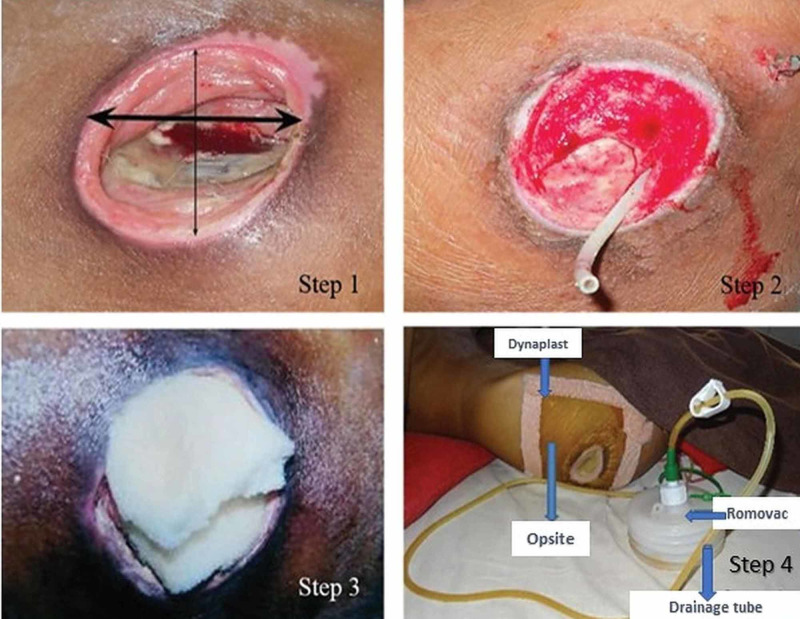
Steps of application of the novel negative pressure device Step 1: The length and width of the ulcer are marked and the surface area is measured with a centimeter ruler. Step 2: One end of the drainage tube of Romo Vac is placed on the ulcer bed. Step 3: Sterilized foam is placed on top of the wound. Step 4: The ulcer is covered using Opsite with an airtight seal and the other end of the drainage tube is pierced inside out 10 cm away and connected to the Romo Vac.

Application of the NPD and all subsequent dressing-related procedures occurred at the patient’s bedside. The perforated end of the drainage tube of the Romo Vac was placed on the wound surface and its other end exited through the skin 10 cm away from the wound margin and was connected to the Romo Vac bellow. The sterilized foam was trimmed according to the size and geometry of the wound and placed on top as a cover. Opsite finally covered the wound and the adjoining healthy skin with an airtight seal. The bellow of Romo Vac is charged to attain appropriate cyclical/intermittent negative pressure (Figure 1). The pressure was measured by a pressure monitoring device. Patients and caregivers were taught how to charge the Romo Vac and advised to charge it after every five to six hours. The NPD dressing was changed weekly or more often if the dressing became saturated or loss of suction occurred. The NPD dressing was changed by the same investigator throughout the study. All investigators received education on the use of the NPD.

Outcome measures

The outcome measures were length, width (surface area), and depth of the PU, exudates (by visual inspection according to the PUSH tool) and tissue type (necrotic tissue, slough, and red granulating tissue) from zero- to nine-week follow-up. Data were recorded every seven days and analysed at weeks 3, 6, and 9 in both the groups.

Data analysis

The Statistical Package for the Social Sciences, version 21 (IBM Corp, Armonk, NY) was used for all data analyses. Descriptive findings were characterized as means ± standard deviations, and group comparisons were completed via the Student t-test. For variables that were not continuous or not normally distributed, we used a nonparametric equivalent to the Student t-test, the Mann-Whitney U test. The mixed linear model was used to find the changes from baseline to week 9. A fixed and random-effects model was used. A p-value <0.05 was considered significant.

## Results

To compare the two therapies, the rate of healing was measured in terms of reduction of surface area and depth of PUs at different follow-up points. There was no significant difference in the length of PUs between the groups till week 3. A significantly reduced length of PUs in the NPWT group was observed at week 6 (p=0.04) which further reduced at week 9 (p=0.001) as compared to the standard wound care group. Similarly, a significant reduction in the width and depth of PUs was observed in the NPWT group at week 9 (p<0.05) as compared to the standard wound care group (Table 1).

**Table 1:** Comparison of length, width, and depth between two groups NPWT, negative pressure wound therapy. Values are presented as means±SDs. *p<0.05 is considered as statistically significant.

Time period	Surface area								
	Length			Width			Depth		
	NPWT group	Standard wound care group	p-value	NPWT group	Standard wound care group	p-value	NPWT group	Standard wound care group	p-value
Week 0	7.46±2.03	7.16±2.17	0.64	5.53±1.65	6.31±2.27	0.70	5.75±1.38	5.31±0.76	0.07
Week 3	4.55±1.92	5.32±1.70	0.16	3.63±1.29	4.63±1.93	0.07	3.24±1.38	2.85±0.52	0.12
Week 6	3.05±1.91	4.23±1.85	0.04*	2.57±2.12	3.51±1.67	0.11	1.84±1.15	1.95±0.46	0.68
Week 9	1.51±1.61	3.25±1.65	0.001*	1.19±1.31	2.46±1.72	0.006*	0.61±0.85	1.16±0.51	0.01*

The volume of exudate was assessed by visual inspection when dressing was removed and categorized as none, light, moderate, or heavy. Exudate levels were significantly (p=0.001) lower in the NPD-treated group as compared to standard wound care group from week 3; this difference continued through week 9 (p=0.001). The disappearance of slough (dead yellowish tissue) and formation of healthy red granulation tissue was significantly higher in the NPD-treated PUs after week 6 (p=0.001) that continued at week 9 (Table 2).

**Table 2:** Comparison of exudate and granulation tissue between two groups Values are represented as means±SDs. ^1^Unpaired t-test. *Significant (p<0.05).

	Exudate			Granulation tissue		
	NPWT group	Conventional group	p-value^1^	NPWT group	Conventional group	p-value^1^
Week 0	3.10±0.30	3.04±0.20	0.51	3.10±0.30	3.21±0.45	0.17
Week 3	2.61±0.49	2.91±0.21	0.001*	2.76±0.35	3.01±0.01	NA
Week 6	1.52±0.68	2.17±0.49	0.001*	2.00±0.31	2.51±0.53	0.001*
Week 9	0.16±0.37	1.25±0.76	0.001*	1.01±0.31	2.05±0.37	0.001*

## Discussion

PUs are complex and chronic wounds in patients with SCI and no gold standard has yet been established for their prevention and treatment. PUs are difficult to prevent and manage and can lead to a decline in the overall well-being of patients in SCI [2,4,5,16]. In this study, the novel NPD proposed by us showed a statistically significant improvement in PU healing in terms of reduction in the surface area and depth, slough clearance, granulation tissue formation, and removal of exudate as compared with standard wound care. The direct costs to treat a PU by our NPD were low as compared to conventional dressing methods. The total cost of a nine-week treatment of one PU in the NPWT group was approximately 47% less than the costs of the conventionally treated comparable PU. Therefore, our device is financially viable and effective in settings where resources are limited.

In this study, for removing the potential risk of bias, a standard procedure was followed for pressure offloading by application of an air tube ring and nursing of the patients, including turning of body, positioning, and bladder and bowel management. A loss of sensation in the skin, constant pressure, moisture, and irritation to the skin in traumatic paraplegia subjects further delay the healing process [17]. Besides many dressing modalities and techniques available for wound healing, an enormous effort is required for the management of these wounds. Recent interest in the treatment of chronic wounds has shifted from the type of dressing with or without pharmaceutical agents to different therapies. Management of pressure ulcers is an ongoing clinical challenge for health care professionals. The conventional methods of treatment require prolonged hospitalization with considerable morbidity in terms of pain, discomfort, and economic burden.

NPWT is a recent technical innovation in wound care with a growing number of applications. In NPWT, the application of topical negative pressure (TNP) on wound bed removes blood and serous fluid, may reduce bio-burden, and increases localized blood flow thereby supplying the wound with oxygen and nutrition to promote accelerated healing [12]. While NPWT is effective in promoting wound healing, the molecular and physiological mechanisms associated with this therapy are still under investigation and not entirely defined. In earlier studies on NPWT, the researchers have concluded that this technology should be considered "the treatment of choice" for chronic (hard-to-heal) ulcers because of its significant advantages of time for wound healing and "wound bed preparation" compared with conventional therapy [9,18]. Ashby et al. in 2012 conducted a pilot randomized trial with 12 patients: six with NPWT and six with standard wound care. In this study, PU healed completely (NPWT group) during the follow-up [17]. Our experience shows that treatment with NPWT could be used as a manageable method in primary care for treating PUs.

A systematic report published in 2015 demonstrated the benefits of a bellows-powered NPWT device designed specifically for use in resource-constrained settings. They found that the elimination of air leaks in the simplified NPWT dressing is essential and that their system is safe and feasible for use in these environments. Application of proven therapies such as NPWT in resource-constrained settings is limited by cost and lack of electrical supply [1]. Subsequent trials will study the system's efficacy. The available commercial devices for negative pressure provide rental services to the patients and the consumable for the device is much more expensive. To provide an alternative to existing electrically powered NPWT systems, our bellows-powered indigenized NPD was safe, easy to apply, and cost-effective in the management of pressure ulcers in traumatic paraplegia subjects.

PU treatment with our novel device (NPD) led to accelerated healing in the majority of cases. Our study shows that PU treatment with our NPD can be used as a manageable method in primary care settings such as home care. This device provides negative pressure of −125 mm Hg pressure (−60 to −125 mm Hg) when fully charged. With time, the negative pressure is gradually lost, requiring periodic manual recharge of the device. This by itself provides an intermittent negative pressure as suggested by Morykwas et al. [9]. They compared the commercial V.A.C.® (3M + KCI, San Antonio, TX) device to standard wound dressings on acute wounds in animal models and reported that negative pressure (−125 mm Hg) improved wound blood flow, particularly after intermittent cessation of pressure.

Mody et al. in 2008 conducted a randomized controlled trial comparing a locally constructed topical NPD with wet-to-dry gauze dressings on varied wound etiologies including diabetic foot ulcers, PUs, cellulitis/fasciitis, and other types of ulcers [1]. Except in a sub-set of PUs, the authors did not find any statistically significant differences in the time to closure between the two treatment groups. They found a significant difference in time to closure of PU (mean 10±7.11 days) between the treatment and control groups (27±10.6 days, p=0.05) [1,2,4]. In our study, the reduction in surface area and depth of PUs treated by our NPD was faster than the PUs treated by standard wound care.

We found that the treatment of PU with our innovative device has superior healing as compared to standard wound care with modern dressings while giving clinicians a simpler method. Two prospective non-randomized clinical trials on PUs showed positive results of NPWT on the healing process [4,5]. Our study gave sufficient evidence on the effectiveness of NPWT over standard regimes of wet-to-dry dressings as a control treatment although NPWT and wet-to-dry dressing both may accomplish mechanical debridement and keep wounds moist. A significant reduction in wound surface area was also observed in patients with PUs treated with NPWT [10]. NPWT has also been shown to rapidly reduce wound surface area and volume and may be especially useful with deep wounds like PUs and diabetic foot ulcers [19]. Our data showed that the percent reduction of length, width, and depth of PUs was more in the NPWT group at weeks 6 and 9. Furthermore, the reduction in the depth of PUs was faster than for length and width. This shows that PUs heal primarily from depth followed by length and width. The application of negative pressure over the wound bed allows the arterioles to dilate, increasing the effectiveness of local circulation, promoting angiogenesis, which assists in the proliferation of granulation tissue [4,9]. We observed that the patients on NPWT had the early appearance of granulation tissue as compared to the patients treated by conventional dressings. The NPD allowed optimal wound closure by preparing the wound for skin grafting or flap closure as and when required.

Slough comprises dead white blood cells, fibrin, cellular debris, and liquefied devitalized tissue. NPWT was originally utilized to speed bedside debridement of wounds [1]. We observed the same with our NPD and by the end of week 6, the slough was removed and replaced by red granulation tissue in all NPWT group cases. NPD accelerates PU healing by providing a moist and closed wound environment. Debrided wounds respond to NPD better than non-debrided wounds because devitalized tissues clog the drain tube disrupting the optimal negative pressure.

The negative pressure speeds up the formation of granulation tissue and reduces bacterial counts in the wound [5,6,9]. Our study revealed a statistically significant increase in granulation tissue formation and an improvement in PU healing in terms of slough clearance and a reduction in bacterial count. NPWT is generally well tolerated and with few contraindications or complications and is thus becoming a mainstay of current wound care. The cyclical application of sub-atmospheric pressure alters the cytoskeleton of the cells in the wound bed, triggering a cascade of intracellular signals that increases the rate of cell division and subsequent formation of extracellular matrix fibers followed by red granulation tissue. In this study, significantly, an increased rate of granulation tissue formation was shown to occur with intermittent NPWT application by our innovative NPD.

Wound exudate is produced as a natural and essential part of the healing process. However, the overproduction of wound exudate, in the wrong place or of the wrong composition, can adversely affect wound healing. In the normal wound healing cascade, exudate makes a moist wound environment and supports healing by facilitating the diffusion of vital healing factors (e.g. growth and immune factors) and the migration of cells across the wound bed and prevents the wound from drying out. As healing occurs, the amount of exudate produced usually decreases [5]. It is important to recognize that the volume of exudate is related to the surface area of the wound, and therefore large wounds such as PUs often produce higher volumes of exudates. When the amount of exudate exceeds the optimal level, it disrupts the healing. In our study, the wound exudate (discharge) became significantly lower in the NPWT group than the conventional group at the third week and reduction continued till the sixth week. There was minimal discharge in the NPWT group after the sixth week. This occurs due to continuous removal (suction) of exudate by the negative pressure generated by the NPD. In the conventional method of dressing, there may be not such a mechanism that removes excessive exudate. The exudate that is produced remains present on the wound bed until the dressing has been removed. Excessive exudate on the wound bed delays healing as it contains matrix metalloproteinases (MMPs) that destroy the collagen fibers as well as newly formed red granulation tissue. Furthermore, wound infection may also facilitate excessive exudate production. It is noted that a NPD covers the wound in such a manner that it prevents the ingress of the surrounding air and makes the wound environment aseptic and moist. As healing proceeds, the level of exudate becomes optimal and red granulation tissue formation along with the deposition of collagen fiber begins on the wound bed.

## Conclusions

NPWT may be the future of wound healing. The commercial devices for negative pressure require rental services from the patients and the consumables are expensive. As an alternative to existing electrically powered NPWT systems, our bellows-powered novel NPD was safe, easy to apply, and cost-effective. The patient compliance was good to excellent as the novel NPD was patient friendly and the airtight seal was very effective. Additionally, the sealed system was easy to use that made the patient's and caregiver's experience better. The procedure was also well tolerated by the patients at their home care. The procedure being safe with minimal side effects can be promoted as an OPD procedure with weekly follow-ups for change of dressings, thereby reducing the hospital stay and hence the economic burden on the patient and the hospital.
